# Does characterising patterns of multimorbidity in stroke matter for developing collaborative care approaches in primary care?

**DOI:** 10.1017/S1463423619000240

**Published:** 2019-07-16

**Authors:** Maria Raisa Jessica (Ryc) V Aquino, Grace M Turner, Jonathan Mant

**Affiliations:** 1 Research Associate, Primary Care Unit, Department of Public Health & Primary Care, University of Cambridge, Cambridge, UK; 2 Research Fellow, Institute of Applied Health Research, University of Birmingham, Birmingham, UK; 3 Professor of Primary Care Research, Primary Care Unit, Department of Public Health & Primary Care, University of Cambridge, Cambridge, UK

**Keywords:** integrated care, interprofessional collaboration, multimorbidity, stroke, transient ischaemic attack

## Abstract

Stroke and transient ischaemic attack (TIA) remain leading causes of mortality and morbidity globally. Although mortality rates have been in decline, the number of people affected by stroke has risen. These patients have a range of long-term needs and often present to primary care. Furthermore, many of these patients have multimorbidities which increase the complexity of their healthcare. Long-term impacts from stroke/TIA along with care needs for other morbidities can be challenging to address because care can involve different healthcare professionals, both specialist and generalist. In the ideal model of care, such professionals would work collaboratively to provide care. Despite the commonality of multimorbidity in stroke/TIA, gaps in the literature remain, particularly limited knowledge of pairings or clusters of comorbid conditions and the extent to which these are interrelated. Moreover, integrated care practices are less well understood and remain variable in practice. This article argues that it is important to understand (through research) patterns of multimorbidity, including number, common clusters and types of comorbidities, and current interprofessional practice to inform future directions to improve long-term care.

## Introduction

Stroke is a leading cause of death and disability worldwide with an estimated annual incidence of 16.9 million first strokes and 6 million stroke-related deaths (Krishnamurthi *et al.*, 2013). Although the age-specific incidence of stroke and transient ischaemic attack (TIA) has been in decline globally, the number of people affected by these conditions has increased (Feigin *et al.*, 2017). Based on the Global Burden of Disease 2013 study, stroke is the third leading cause of disability worldwide (Feigin *et al.*, 2017). Furthermore, stroke and TIA patients are at high risk of recurrent stroke (Mohan *et al.*, 2011). The majority of risk factors for these conditions are modifiable (Feigin *et al.*, 2016).

Often, stroke and TIA survivors present with multimorbidity – having at least two or more chronic conditions (Barnett *et al.*, 2012; Wallace *et al.*, 2015). Related to this, comorbidity refers to co-existing conditions with an index condition of specific interest (van den Akker *et al.*, 1996). Although we pay particular attention to stroke/TIA as conditions of interest, for the purposes of this paper we use the term multimorbidity, because stroke/TIA might not be the primary condition of a patient and might present interdependently or independently of other conditions (Barnett *et al.*, 2012; Gallacher *et al.*, 2014; Lefèvre *et al.*, 2014).

Whilst multimorbidity is widely recognised for older adults with a stroke/TIA diagnosis, evidence also shows that many people aged <50 years including stroke survivors (Maaijwee *et al.*, 2014) also experience multimorbidity (Barnett *et al.*, 2012; Cassell *et al.*, 2018). The development of efficient and cost-effective care models to better serve this population remains high on policy agendas (National Heart Stroke and Vascular Health Strategies Group (Australia), 2004; Department of Health, 2007; U.S. Department of Health and Human Services, 2010; Centers for Disease Control and Prevention, 2012; The Scottish Government, 2014; The Ministry of Health and Care Services (Norway), 2015) and reinforced in the World Health Organization’s (2013) Global Action Plan for the Prevention and Control of Noncommunicable Diseases. Integrated care approaches to managing multimorbidity, often involving different health and social care professionals, are encouraged particularly in primary healthcare, where the majority of care is provided and needs are addressed (Roland *et al.*, 2012; Kringos *et al.*, 2015; Cassell *et al.*, 2018).

A better understanding of both stroke and TIA multimorbidity and current provisions for managing these could inform the development of sustainable care models in the future. The role of primary healthcare is important in this context, as re-emphasised by the 40^th^ Anniversary of the Alma-Ata Declaration. In the draft Declaration 2.0 for 2018, primary healthcare services ought to: offer comprehensive, well-coordinated, long-term care that is people centred and responsive to their context and needs (World Health Organization, 2018). Therefore, this article aims to discuss the current literature on multimorbidity in stroke/TIA and the contemporary challenges to managing multimorbidity in relation to these conditions in primary care, and argue the case for research into profiling multimorbidity in relation to stroke/TIA, and characterising integrated care approaches for this population.

## What is known about multimorbidity in stroke/TIA

### Epidemiology

Multimorbidity is common in stroke and TIA (Tran *et al.*, 2018; The Academy of Medical Sciences, 2018). Gallacher and colleagues (2014) collated the evidence for multimorbidity in stroke across 40 conditions in Scotland and found that the overwhelming majority (94.2%) of those with a stroke diagnosis in their GP records (*n* = 35 690) had one or more existing morbidities excluding stroke, whereas, only 48% of the comparator group (ie, no stroke, *n* = 1 388 688) had one or more morbidities. The two most frequent physical comorbidities for those with a diagnosis of stroke were hypertension and coronary heart disease, consistent with other studies (Barnett *et al.*, 2012; Bergman *et al.*, 2015; O’Donnell *et al.*, 2016).

A large-scale study of multimorbidity in cardiovascular disease (ie, ischaemic heart disease, and stroke or TIA) across 56 conditions found that of 4.2 million UK adults, 229 205 individuals experienced cardiovascular disease (Tran *et al.*, 2018). Between 2000 and 2014, rates of multi- and comorbidity increased fourfold (6.3–24.3%) for this population. However, this study did not report the comorbidities associated with stroke/TIA specifically. Also in the UK, Gallacher and colleagues (2018) found, in a sample of 8 751 stroke/TIA patients, patients with multimorbidity have increased risk of mortality and this risk increases as the number of comorbid conditions increases (regardless of type of condition). Both studies found higher numbers of comorbidities in females, older age groups, and people living in deprived areas (Gallacher *et al.*, 2018; Tran *et al.*, 2018). Comorbid conditions are also associated with poorer functional outcome post-stroke (Karatepe *et al.*, 2008). However, pairings or clusters of comorbid conditions – types of co-occurring conditions – in relation to outcomes other than mortality, such as quality of life, were not explored.

The differences between the temporal relationship (ie, pre-stroke, post-stroke, unrelated) and timespan in which comorbid conditions present in relation to stroke/TIA also need further exploration given subsequent implications on treatment, care management, and health service financing and planning (Valderas *et al.*, 2009). One study found an association between stroke and subsequent increased risk of progression to heart disease and diabetes; however, the population was women only and self-report data were used (Xu *et al.*, 2018). A nuanced understanding of cardiometabolic and non-cardiometabolic comorbidities could help with developing strategies that better take these into account.

### Measures of multimorbidity

Measuring multimorbidity is challenging due to a lack of international consensus regarding its conceptualisation, scope, and how conditions are defined. Comparison of multimorbidity prevalence rates across different populations and settings is hindered by availability of data, the variety of measurement tools, and the broad range of conditions included in existing multimorbidity measures (Fortin *et al.*, 2012; Lefèvre *et al.*, 2014).

A recent study concerning the key factors for consideration when measuring multimorbidity concluded that there is ‘no single “best” measure of multimorbidity (p. 6)’ (Griffith *et al.*, 2018). Broadly, multimorbidity measures are characterised as non-weighted (ie, frequency counts of co-occurring diseases) or weighted indices (ie, accounting for condition severity, healthcare utilisation) (Huntley *et al.*, 2012). The most commonly used multimorbidity measures are disease counts, weighted measures particularly the Charlson Comorbidity Index, the Cumulative Illness Rating Scale, the Index of Coexistent Disease, and the Adjusted Clinical Groups System (Johnston *et al.*, 2019). However, in stroke epidemiology research, frequency counts are most commonly used (Gallacher *et al.*, 2014; Lefèvre *et al.*, 2014; Bergman *et al.*, 2015; Gallacher *et al.*, 2018; Tran *et al.*, 2018; Xu *et al.*, 2018).

Whilst these indices are useful for a range of purposes (eg, clinical, research, health services planning), some are decades old (Linn *et al.*, 1968; Charlson *et al.*, 1987) and conditions included in these measurement tools might not reflect the contemporary conditions that are known to increase the risk of mortality. Furthermore, some measurement tools have been developed using a limited population (Charlson *et al.*, 1987). A recent expert panel study has identified that current multimorbidity measures do not account for social determinants of health and mental health, and episodic conditions (ie, recurring conditions or previous diagnoses that have already been treated). Such factors are important to consider when developing models of care and health policy (Griffith *et al.*, 2018). Therefore, a challenge for multimorbidity research is capturing the complex nature of multimorbidity – going beyond physical diagnoses – and ensuring that the appropriate measurement tools are a good fit with the study purpose and/or stakeholder objectives.

### Managing multimorbidity in relation to stroke/TIA

The evidence for increasing multimorbidity in stroke/TIA has implications for treatment burden, including but not limited to polypharmacy (Ostwald *et al.*, 2006; Gallacher *et al.*, 2014), healthcare utilisation (Cassell *et al.*, 2018), and treatment adherence (Mair and May, 2014; Tran *et al.*, 2018). Importantly, there is evidence demonstrating that variations in multimorbidity patterns across different age groups (Tran *et al.*, 2018) and socioeconomic backgrounds (Bray *et al.*, 2018; Gallacher *et al.*, 2018) have profound and wide-ranging impacts on people’s functioning, well-being, and quality of life (Fortin *et al.*, 2004; Navickas *et al.*, 2016). For example, people living in areas of deprivation tend to have a first stroke earlier (Bray *et al.*, 2018) and are at greater risk of experiencing comorbidities (Barnett *et al.*, 2012), when compared to their more affluent peers.

Stroke is a leading cause of disability and many patients have complex needs, including physical, emotional, social, communication, and cognition needs (Stroke Association, 2018). Post-stroke care often requires complex management plans to address patients’ rehabilitation, social care, and stroke prevention needs. Beyond the array of impacts strokes/TIAs can have on individuals, their experiences of primary healthcare present further challenges to service delivery. A qualitative systematic review of patients’ experiences of stroke management describes post-stroke ‘treatment burden’ which is intensified by poor communication and fragmented healthcare (Gallacher *et al.*, 2013). Additionally, a systematic review with meta-ethnography by Pindus and colleagues (2018) showed that often stroke survivors and their carers felt marginalised and abandoned due to the passivity of services. This passivity was characterised by constraints to access to care, limited continuity of care, poor communication between healthcare professionals as well as between providers and stroke survivors and their carers, and receiving varied information about stroke (Murray *et al.*, 2003). These are interacting factors that make caring for patients with multimorbidity a challenge to healthcare providers and systems (Mair and May, 2014).

Multimorbidity is likely to exacerbate this post-stroke treatment burden through multiple uncoordinated appointments, polypharmacy, and lack of continuity of care (Noël *et al.*, 2005). Multimorbidity can also impact on patients’ rehabilitation and recovery; for example, comorbid knee arthritis impairs patients’ ability to fully engage in stroke rehabilitation which can manifest in frustration and required additional coping strategies (Wood *et al.*, 2009). Similarly, anxiety and depression have been found to slow recovery from stroke (West *et al.*, 2010). Stroke patients with comorbidities are often excluded from rehabilitation clinical trials (Nelson *et al.*, 2017); therefore, evidence-based interventions and recommendations might not be appropriate for the large proportion of stroke patients with comorbidities.

Despite multimorbidity being commonplace, clinical guidelines remain, by and large, disease specific and can be potentially harmful to patients and burdensome for healthcare professionals (Boyd and Fortin, 2010; Parekh and Barton, 2010; Guthrie *et al.*, 2012). Integrated care approaches for people with multimorbidity can offer opportunities to treat conditions that have common management strategies together (eg, hypertension and coronary heart disease, risk factors for stroke) including mental health comorbidities that could co-occur with physical conditions. However, a recent large-scale cluster-randomised controlled trial in GPs in England and Scotland tested a 3D approach to care (ie, a patient-centred way of managing dimensions of health, depression, and drugs) of patients with multimorbidity found no improvements in health-related quality of life. Nevertheless, the study found that such an approach enhanced patient-centred care, including patient satisfaction (Salisbury *et al.*, 2018). To date, the evidence for integrated care in primary care for people with multimorbidity is equivocal (Smith *et al.*, 2012; Salisbury *et al.*, 2018) and requires further investigation particularly in relation to stroke/TIA. Future studies testing complex interventions such as that of Salisbury *et al.*’s (2018) should include process evaluations, where feasible and cost effectiveness analysis if the findings are positive (Ramsey *et al.*, 2005; Moore *et al.*, 2015). Furthermore, an understanding of the temporal relationship between conditions offers the opportunity for preventative interventions, for example, to prevent the cluster of diabetes, heart disease, and stroke (Xu *et al.*, 2018).

Multimorbidity care practices vary and could be broadly classified into three groups: (i) those that are focussed on a specific disease (ie, ‘index disease’) and include other conditions as comorbid; (ii) those that are focussed on specific combinations of a number of chronic diseases; and (iii) those that are not confined to specific combinations of chronic diseases (Rijken *et al.*, 2018). Moreover, although interprofessional collaboration is a reported key feature of integrated care models (Lalonde *et al.*, 2012; Valentijn *et al.*, 2013), a multitude of terms are used interchangeably in integrated care and interprofessional collaboration literature (Atwal and Caldwell, 2002), making our understanding of how care pathways and collaborative care practices are implemented in practice to address multimorbidity in stroke/TIA a challenge. As such, the range of approaches to managing multimorbidity in primary care can also introduce gaps in care and service delivery, with knowledge concerning best practices and health organisation remaining limited (Rijken *et al.*, 2018; The Academy of Medical Sciences, 2018). These require illumination, as identifying markers of successful integrated care and interprofessional collaboration can impact care and professional practice.

Few randomised controlled trials have investigated the effectiveness of interventions to enhance multimorbidity management; nevertheless, the available literature has focussed on interventions geared toward changes to the organisation of care or enhancing interprofessional collaboration (Smith *et al.*, 2012; Salisbury *et al.*, 2018). Given that stroke and TIA survivors frequently seek medical support through primary care, it is imperative that current practices, particularly in the context of multimorbidity, are better understood. This is in line with the shift toward integrated care models, which involve multiple healthcare professionals working together to address multimorbidity, such as GPs, district or community nurses and pharmacists (Rijken *et al.*, 2018). A particular challenge is determining which of these integrated care approaches are predominantly applied (including where and how), which are most effective, and which are most acceptable to stroke/TIA patients experiencing multimorbidity.

### Going forward

Considering our current knowledge of multimorbidity in stroke/TIA and how these are managed, we outline several suggestions for advancing our understanding of these. First, a more detailed understanding of multimorbidity in stroke/TIA is needed. Specifically, pairings and/or clusters of multimorbidity in stroke/TIA need to be identified, and their relationships with and impact on clinical and patient-reported outcomes assessed. Understanding the impact of multimorbidity on stroke patients’ rehabilitation and recovery is necessary to improve health and social care post-stroke. Measures of multimorbidity and their burden are heterogeneous and thus have implications on how associations and outcomes are investigated, which need to be considered in relation to available data (Huntley *et al.*, 2012).

Studies of multimorbidity trends in stroke/TIA need to select measures of multimorbidity based on study purpose and stakeholder groups involved. Continuity of care, treatment burden, and patients’ experience could potentially be improved by efficient exchange of information between interdisciplinary healthcare providers, particularly specialists, GP, and the third sector. However, multiple barriers for optimal communication exist, such as lack of integration of electronic health records (Sadler *et al.*, 2017). New models of care are required to address poor communication between healthcare providers to improve transition of care and follow-up for stroke/TIA patients in the context of multimorbidity.

Importantly, multimorbidity trends in stroke/TIA need to be explored in different contexts (eg, urban versus rural communities) in different countries (ie, high-income versus low- and middle-income), as well as different care settings (ie, primary care versus secondary care) to contribute to the ‘global atlas’ of multimorbidity (The Academy of Medical Sciences, 2018). Future research should explore the settings in which multimorbidity in stroke/TIA is managed. This needs to be better understood, especially in low- and middle-income countries, as multimorbid conditions have implications on healthcare resources allocation and patient-centred care (Valderas *et al.*, 2009). Indeed, the vast majority of the multimorbidity literature is derived from high-income countries, despite most of non-communicable diseases occurring in low- and middle-income countries (Xu *et al.*, 2017). Specifically, the definitions, components, provider profiles, and quality indicators of integrated care approaches for stroke/TIA need to be better characterised. The evidence base for multimorbidity management and risk factors need to be built alongside epidemiological evidence on multimorbidity in low- and middle-income countries, which is limited (Xu *et al.*, 2017). A further step would be to relate these integrated care approaches to health outcomes (eg, quality of life) and assess these approaches’ cost-effectiveness, which will require more prospective, longitudinal research. Extensive research output from high-income countries can inform future research in low- and middle-income countries (Xu *et al.*, 2017). For example, the influence of social determinants of health on multimorbidity rates in these contexts should be explored. The burden of both communicable diseases and non-communicable diseases on multimorbidity should be assessed, particularly as communicable diseases remain a challenge in many countries.

### Concluding thoughts

The number of stroke and TIA survivors has been increasing, and with this increase comes multiple challenges – including multimorbidity – to the healthcare system especially primary healthcare services. A more considered understanding of the combinations of diseases that co-occur with stroke/TIA in terms of how these vary across different patient subgroups living in different contexts and their associations with clinical and patient-reported outcomes serve the important purpose of: (i) identifying their risk factors for developing further morbidity, and/or experiencing another stroke or TIA and (ii) developing context-appropriate strategies for addressing wide-ranging and long-term needs, such as cognitive impairment and rehabilitation. From this understanding, new models of integrated care practices can be developed, informed by evidence and current effective integrated care practices. The landmark Alma-Ata Declaration’s 40^th^ anniversary brings to the spotlight the need for high-quality, integrated primary healthcare in the context of an ageing population and the rise of multimorbidity globally. Patient-centred, individualised, and well-coordinated care is required to optimise stroke recovery and reduce treatment burden in relation to patients’ morbidities. There is a tension within longer term stroke care as to the extent to which this is best delivered by generalists and the extent to which it should be delivered by specialists. A more nuanced understanding of the epidemiology of multimorbidity in stroke/TIA will help inform this debate.
